# Interactions Among Cross-Border Contiguous Communities and Implications for Managing Pandemics – The case of Ghana and Burkina Faso During the Ebola Outbreak in West Africa: A Qualitative Study

**DOI:** 10.5334/aogh.3807

**Published:** 2022-05-30

**Authors:** Dennis Chirawurah, Niagia Santuah, Stephen Apanga

**Affiliations:** 1 University for Development Studies, School of Public Health, Department of Environmental and Occupational Health, GH; 2 University for Development Studies, School of Medicine, The West Africa Resilience Innovation Lab, GH; 3 University for Development Studies, School of Medicine, Department of Community Medicine, GH

**Keywords:** Cross-border, Contiguous communities, Ghana, Burkina Faso, Ebola Virus Disease

## Abstract

**Introduction:**

In sub-Saharan Africa, extensive migratory activities and interactions exist especially amongst unmanned cross-border communities between countries sharing common borders which complicate emergency public health interventions. Understanding the nature of these activities and interactions will help strengthen public health interventions and control of pandemics such as the Ebola outbreak and COVID-19.

**Objective:**

The study aimed to understand the nature of contiguous border communities’ interactions and to seek community solutions for building efficient and resilient health systems to combat a possible Ebola outbreak in Ghana and Burkina Faso and the control of future pandemics.

**Methods:**

A qualitative cross sectional study design using focused group discussions and key informant interviews involving six focused groups and forty-six key informants were conducted amongst six Kasem-speaking contiguous border communities, three-each in Ghana and Burkina Faso.

**Findings:**

Findings of interactions consisted of social interactions such as marriage ceremonies; traditional and religious practices; informal trade; and health seeking behavior in the study communities. Collaborative disease surveillance systems; constructive dialogue involving community traditional leaders; incorporation of health education into social, traditional and religious activities; retraining of health personnel; effective communication including networking; and inter-governmental collaborations were identified as solutions to the effective control of the Ebola outbreak and for future public health interventions in general.

**Conclusion:**

Understanding community interactions and seeking community solutions were identified to be crucial in building efficient health systems that are resilient and responsive to the Ebola outbreak and for future pandemics in contiguous border communities in sub-Saharan Africa.

## Introduction

The interconnected and cross-border nature of countries sharing common borders in sub-Saharan Africa facilitates the transmission of epidemics and pandemics which is often further compounded by the very porous nature of their borders [1,2,3]. The case of the outbreak of the Ebola virus disease (EVD) typifies this challenge. What started as a public health crisis in Guinea on 26 December 2013 degenerated into a development crisis in the three epicentre countries of Guinea, Liberia and Sierra Leone in less than six months. By December 2014, the number of EVD cases in this outbreak was four times higher than the combined total of all prior outbreaks reaching 22 859 cases and 9 162 deaths in all three countries as of 11 February 2015 [4].

The Ebola outbreak attained a global dimension in October 2014 when health workers in Spain and the United States contracted EVD while providing care for Ebola patients, which further exposed the urgent need for global action against the virulent disease [1,4]. Containing the epidemic in the three most affected countries was therefore necessary and its substantial impact on all West African economies was already anticipated.

Formal and informal migratory activities exist along the borders of cross-border communities with the main drivers of these movements being economic, linguistic, familial, health-seeking, cultural and social networks amongst others leading to the diffusion of epidemics [1,3,5]. These activities not only challenge national systems’ capacities to detect diseases of global public health interest but also undermine the implementation of the revised World Health Organization’s (WHO) International Health Regulations (2005) (IHR) which require governments to inform WHO of all events within their territory that may constitute a public health emergency of international concern [3,5,6,7].

Despite the official closure of borders between Ghana and Burkina Faso during the Ebola outbreak in West Africa, migratory interactions amongst contiguous or frontline cross-border communities continued mainly through unmanned and unapproved routes thereby undermining official border closure as an effective epidemic control measure between these two countries. Understanding the nature of these interactions will help build resilient health systems and structures to strengthen public health interventions and stave off pandemics.

The aim of this study was to understand the nature of frontline cross-border communities’ interactions and to seek community solutions or contributions towards building resilient public health systems to contain a possible Ebola outbreak in both Ghana and Burkina Faso and for the control of future pandemics.

## Materials and Methods

### Study setting

The study was conducted in contiguous communities in the Kassena-Nankana West District in the Upper East region of Ghana and the Commune Urbaine de Pô and the Commune de Tiébélé, both in the Nahouri Province of the Centre-Sud region of Burkina Faso. The terms “Region” and “District” in the Anglophone setting (Ghana) approximately correspond respectively to the terms “Province” and “Commune/Départment” in the Francophone setting (Burkina Faso).

The Kassena-Nankana West District is one of the thirteen districts in the Upper East Region of Ghana and shares borders with Burkina Faso to the north. Its population according to the 2010 Population and Housing Census is 70 667 representing about seven percent of the region’s total population. Seventy-nine percent of the population is rural and engaged in various economic and social activities including; subsistence farming, business/trade, rearing domestic animals among others [8]. The Nahouri province on the hand is one of the 45 provinces in Burkina Faso and located in the Centre-Sud administrative region sharing borders with Ghana to the south. In 2006, the province had a population of 155 463 people. The main ethnic group is the Gurunsi, who are known as the Kassena across the border in the Upper East Region of Ghana. The landscape has few natural barriers and the traditional economic activities of shifting cultivation, semi-nomadic pastoralism and trade is often associated with some degree of migration [9].

**Figure 1:**
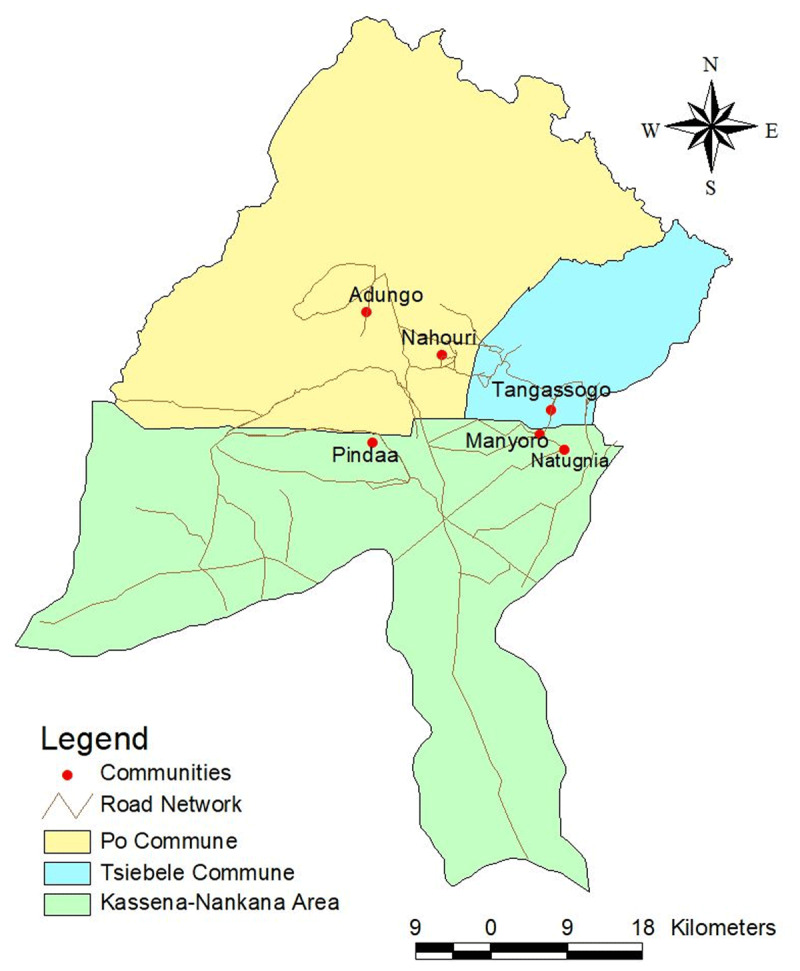
Map of Frontline Border Communities in Southern Burkina Faso and Northern Ghana. Source: Author’s.

The selected communities for this study were Natugnia, Manyoro and Pindaa from Ghana and Adungo, Nahouri and Tangassougou from Burkina Faso (Figure 1). These contiguous or frontline border communities were purposively selected based on the researchers’ linguistic ability and convenience. Besides during a stakeholder engagement, these communities were also identified to have many unapproved routes through which cross-border activities were conducted. They data collection lasted from 1^st^ June to 31^st^ July, 2015. We used the first two weeks to visit the study areas and held meetings with local government functionaries, community traditional rulers and community opinion leaders to explain the objectives of the study and to elicit their support and cooperation.

### Study design

This was a qualitative cross sectional study using interviews. The interview, described as “a device for inciting narrative production” [10] is an important way of collecting qualitative data [11,12]. This is based on the traditional view to regard the person being interviewed (the respondent) as a “repository of information” or a “basket full of answers” [10]. This study used two different kinds of interviews: Focus Group Discussion and Key Informant Interview. A Focus Group Discussion (FGD) enables researchers to hold conversations among groups of people purposely chosen because they possess narrative competence regarding a specified research topic. Because these conversations take place in the language of the individuals involved, FGDs are very productive for the analysis of perceptions, attitudes, motives, feelings and beliefs about complex issues [13].

Key Informant Interview (KII) is an in-depth interview that collects information from individual experts. This requires careful selection of subjects to gather input from individuals considered to be the most knowledgeable people on the subject. KIIs, whether structured or unstructured, allow the researcher to probe respondents and guide the interview according to their answers [14]. In our study, in addition to earmarking community traditional leaders for in-depth interviews, Key Informants were identified from focus group discussants that demonstrated in-depth knowledge in the thematic areas of the study.

### Participant selection

Participants for the KIIs and FGDs for the study were selected based on a predetermined criterion that included but not limited to: traditional authorities, religious leadership, community opinion leadership, women’s networks, political representatives, health workers, civil society organizations, border security, trans-border transportation unions and traders. All participants were above 18 years.

### Data collection

Two experienced field research teams were composed comprising three research assistants each from both Ghana and Burkina Faso. Some thematic areas were agreed on by the main research team a priori after which the field research teams were then trained on the study protocol, data collection tools including its explanation in the local kasem language and comportment during data collection. The data collection tool for the KIIs was a semi-structured questionnaire with specific questions related to the nature of cross-border interactions; proposed solutions to combating the EVD and control of pandemics; and any other additional information. The data collection tool for the FGDs was an interview guide with questions on the nature of frontline cross-border community interactions and the community’s contribution towards preventing an outbreak of EVD and other pandemics. The data collection tools were written in English for Ghana and translated into French for Burkina Faso by one of the investigators (NS) who is a language expert. However, all interviews were conducted in the common cross-border local language of Kasem and audio-recorded for later transcription after pre-testing the tools in one community each in both countries. Daily field notes were also taken by the research assistants, which were used in the data analysis. A total of 46 KIIs and 6 FGDs (three in each country) were conducted from both the Kassena-Nankana West District and Nahouri Province in Burkina Faso. The average number of participants in the FGDs was 10 for each country.

### Data analysis

The audio-recordings were transcribed verbatim from the local language (Kasem) into English in a text form for analysis. Manual data analysis was done using the framework analysis approach in qualitative data analysis for applied policy research as outlined by Jane Ritchie and Liz Spencer [14,15]. This approach conveniently allows for both the KIIs and FGDs to be analyzed together. Three experienced qualitative research investigators (DC, NS and SA) were involved in the data analysis after agreeing on certain thematic areas a priori. Two (DC and NS) investigators then independently analyzed the data by the initial thematic areas and further generated additional thematic areas and extracted quotes after reading through all the transcripts exhaustively to check for inconsistencies. All three investigators then met together to review the initial analyzed data and where there was a disagreement between the two initial investigators, the third investigator (SA) made the final determination.

### Ethical considerations

The Navrongo Health Research Centre Institutional Review Board of the Ghana Health Services gave ethical approval for the study to be conducted (ID: NHRCIRB 183). Verbal consent was also obtained from the key informants and participants of the FGDs before interviews were conducted.

## Results

### Characteristics of participants

In total, 46 key informants were interviewed with an average age of 51 years, whilst 6 FGDs were conducted with the average age of discussants being 50 years. Table 1 shows the other characteristics of participants involved in this study.

**Table 1:** Characteristics of key informants and focus group discussants.


CHARACTERISTICS	KIIs	FGDs

Gender	Number (%)	Number (%)

Male	26 (57)	37 (56)

Female	20 (43)	29 (44)

Literacy level		

Literate	17 (37)	17 (26)

Illiterate	29 (63)	49 (74)

Total	46 (100)	66 (100)


### Thematic areas

The main thematic areas identified are presented in two sections comprising of the nature of cross-border interactions and its implication for Ebola outbreak; and communities’ solutions to contain a possible Ebola outbreak. The major themes of the nature of cross-border interactions and its implication for Ebola outbreak included social interactions; traditional and religious practices; informal trading; and seeking health care whilst that of communities’ solutions to contain a possible Ebola outbreak comprised of health education and promotion; collaborative efforts; and capacity strengthening for Ebola control.

### Nature of Cross-Border Interactions and Its Implication for Ebola Outbreak

#### Social interactions

Participants identified social interactions between front-line border communities as one of the main forms of interactions. About 90% of key informants in the Nahouri province said social interactions were one of the main cross-border activities whiles 57% of their Kassena-Nankana West District counterparts saw this to be one of the cross-border activities. One key informant is noted to have said;

*…“In our society, community markets are more than concentration centres for the buying and selling of food stuff, livestock and ingredients. It is here we find spousal mates, identify and reconnect with lost family relations and socialize during the dry season. We also believe that it is in the markets our community ancestral spirits and ‘tangonna’ (deities) meet to prevail over happenings in the community” (Community elder at Nahouri – KII)*

Similarly during the FGDs participants identified this to be one of the main cross-border activities with one discussant saying;

*…“We are one and the same people – we intermarry, we have relatives across the border and we also make friends through so many different meetings like markets, funerals and festivals. In the past we go across as a community to weed on our in-laws farms. Our common language, livelihoods and location circumstances tie us all together. If I have my auntie’s children in Navoro Pungu and there is a funeral there, how can the government say that I need a permit to visit them? We easily will take the foot path and go there” (Community elder at Nahouri – FGD)*

#### Traditional and religious practices

One feature that brought people of communities together was their common traditional belief and practice system. Ancestral veneration and soothsayer consultations were a predominantly discussed issue amongst participants. Most (80% from Nahouri Province and 73% from the Kassena-Nankana West District) of the key informants believed that their common traditional heritage constantly brought them together. Speaking strongly on this subject, a key informant contended that;

*…“One major area is our belief in ancestral veneration and following of that path. Many people from Kasem- and Nankam-speaking communities around here come all the way here and go as far as to Gongo in Chebele to consult soothsayers who they believe can ‘see’. Up till date we still witness people come to community acclaimed soothsayers to consult them on their life challenges” (Male key informant at Tangassougou – KII)*

Another frequently mentioned traditional practice identified by participants that is believed to bring people together is the performance of funerals. Participants from the FGDs opined that since Kassena in Ghana and those in Burkina Faso have a common ancestry but only separated by a physical colonial boundary; they still continue to perform their funerals together as one people. These kinds of interactions pose the greatest dangers to the transmission of EVD. One of the participants in a focus group discussion stressed the importance of burial ceremonies among the Kassena people by saying;

…“*When an elder dies, it is required that we organize a befitting celebration of his life and send him to his father’s properly. The ‘bayee’ (undertakers) will carry him in the traditional burial mat whilst dancing, accompanied by war dancers. It is pride of every ‘baya’ (undertaker) to be the one to hold the dead body and lower it into the narrow tunnel grave. Some will even attempt to lie in the grave with the dead body as a sign of the potency of their powers as ‘baya’” (Male participant at Adungu – FGD)*

#### Informal trading

The study gathered from key informants that informal trading activities were very common between communities from both sides of the border mainly through unapproved routes. Some of the trade activities they mentioned included poultry, cloths, motor cycles, food stuff and smuggling of fuel. As one key informant puts it;

*…“You need to come here late night to witness the number of youth involved in carrying petrol and diesel from Ghana through bush paths on motorbikes and cars to sell to their counter parts across the border. It’s a major source of income for most youth in frontline border communities and involves multitudes of people. Petrol is cheaper in Ghana than in Burkina Faso” (Female key informant at Pindaa – KII)*

#### Seeking health care

All key informants from Nahouri Province said that because Navrongo (which is in the Kassena- Nankana Municipal Area and just a few kilometers away from the Kassena-Nankana West District) has a big hospital, people of the border communities from the Nahouri Province cross over to the hospital to seek health care when they are critically ill. On the other hand just under half of key informants (46%) from the Kassena-Nankana West District said people from their border communities cross over to neighboring Burkina Faso to seek health care mainly in traditional medicine and spiritual healing. FGDs participants also reported similar observations. A participant in one of the FGDs asserted that;

*…“There are many traditional ‘healers’ and ‘seers’ in Chebele, Kayaa and Nobre that people travel from near and far to come and consult. People from here with relations across the border in Ghana now have access to the Ghana health insurance cards which enables them to seek medical care from the War Memorial hospital in Navrongo when they are seriously sick” (Female participant at Nahouri – FGD)*

### Solutions to Contain a Possible Ebola Outbreak

#### Health education and promotion

Participants from both KIIs and FGDs were of the view that continuous education to empower people in the communities with adequate knowledge on the Ebola virus disease and its prevention and many other disease outbreaks such as cerebrospinal meningitis should be pursued. One participant in a FGD had this to say.

*…“The first thing is having relevant knowledge through training and learning. We will want to prevent the disease but if we lack knowledge how can we do it?” (Male participant at Tangassougou – FGD)*

Key informants and focus group discussants suggested that health educational and promotional messages and packages should strategically target the hubs of human concentrations such as funeral ceremonies, marriage ceremonies, local markets and other informal trade and traditional healing centers.

#### Collaborative efforts

All study participants saw the need for a collaborative effort to prevent the spread of the Ebola virus in the study areas in Ghana and Burkina Faso. Participants thought it was necessary and important for communities and organizations within each country to work with each other towards the prevention of any emergency Ebola outbreak. One study participant captured the relevance of collaborative effort by saying that;

…“*The nature of the disease is like a force that will compel you to work together in order to avoid it” (Male participant at Pindaa – FGD)*

Key informants particularly saw the need for certain key institutions such as Action Sociale and the Armed Forces from Burkina Faso and the National Disaster Management Organization and the National Centre for Civic Education from Ghana to collaborate in order to effectively control the Ebola virus and any other outbreak. Other institutions that could collaborate for disease control mentioned by the key informants included research institutions, transport unions, Red Cross Society, border security, political administrative agencies, traditional and religious leaderships and the media from both sides.

Key informants suggested pathways for cross-border collaboration to be the inclusion of local government administration leadership, inclusion of frontline border community and traditional leaders, organizing cross-border multi-stakeholder meetings, developing a common cross-border action plan, organizing a common community-based participatory surveillance and having a diplomatic buy-in from Ouagadougou and Accra. It was further suggested that sharing or mobilization of resources (financial, personnel, logistics), effective networking, public education or awareness creation through the formation of a joint task force and peer group educators will also contribute significantly towards Ebola prevention. At the community level, participants suggested communal cleaning of the environment as one way of preventing the Ebola and other disease outbreaks.

However, despite the need for a collaborative effort between both countries, several existential challenges and shortcomings were also expressed by participants. These challenges cut across the social, economic and political realms. Key among the economic realms was inadequate or lack of resources such as logistics (gloves, veronica buckets, nose masks, overalls and soap), personnel and funding. Inadequate training of volunteers and health workers, lack of means for transportation, lack of respect/trust between communities especially Ghana and Burkina Faso were seen as other major challenges. Language barriers, lengthy bureaucracies in accessing information, different political orientations in Ghana and Burkina Faso, lack of political commitment, disunity among people, poor border security systems and poor road network to communities also emerged as challenges.

A clear example of the political component was reflected in one participant lamenting the artificial division of the same people by saying;

…“*Their police and soldiers will interrogate you until you are fed-up such that when they seek for information from you, you feel reluctant to contribute. This is mistrust*” (*Male participant at Manyoro – FGD)*

#### Capacity Strengthening for Ebola Control

The capacity of persons, communities and organizations to effectively deal with disease outbreaks was identified by both key informants and focus group discussants to be very crucial as it underpins the mobilization and judicious utilization of both human and material resources. Although participants alluded to a number of ways through which the capacities of organizations and communities could be strengthened to enable them work together to prevent or respond to possible Ebola outbreak, the training of health workers, volunteers and Red Cross officials on Ebola prevention and treatment was found to be paramount. This was highlighted in the FGD at Nahouri where a participant said;

…*“For the various groups in the communities, if training can be extended to them including the health workers/nurses who are working in the communities, it will be good” (Male participant at Nahouri – FGD)*

Other areas that could enhance the capacity of institutions and individuals in Ebola prevention and response were the creation of emergency centers or committees at the various hospitals, and training of the border security in particular to be able to examine immigrants well. This training, they said should be the same for both countries.

The need to strengthen capacity amongst local community actors through the use of relevant behavioral change communication approaches emerged as one of the effective strategies for controlling the Ebola outbreak. This point was succinctly expressed by one study participant as;

*…“The problem is that the health workers most of the times use information sharing methods that do not help us to completely get whatever message they want us to get. They use posters displayed at selected places and Radio Goulou (FM station) announcements. Although this is done in kasem, at times it is done when we the women are still working and have no time to tune in. However if it is done directly in the community through the traditional rules and community durbars, I think it will help the women understand and ask questions instead of the radio. The deadly nature of what we hear about the Ebola will require them to get to us directly” (Female participant at Adungu – FGD)*

## Discussion

This study aimed to understand the nature of migratory interactions amongst contiguous border communities and their implication for the spread of the EVD and to get local community-based solutions in order to curtail the outbreak and future diseases of global public health interest.

The porous nature of borders associated with cross-border interactions in West Africa have been shown to be responsible for the transmission of diseases. It has been postulated that the interconnected and cross-border nature of the threat facilitated Ebola transmission in West Africa [1,2].

In this study too, human mobility activities existed along the frontline border communities of both Ghana and Burkina Faso which are not part of the official manned border communities between the two countries. Many of these cross-border movements were for various reasons including social such as funeral and marriage ceremonies, informal trade, seeking health care, traditional and religious practices amongst others. These activities have the potential of posing serious risk for the spread of Ebola and other outbreaks. For example, the study revealed that traditional burial rites among the Kassena-Nankana include burial rites being carried out by undertakers who take pride in handling corpses and touching them with their bare hands. This practice is similar to what pertains elsewhere within the sub-region. A WHO report indicated that in West Africa, the Ebola virus spread through the networks that bind societies together in a culture that stresses compassionate care for the ill and ceremonial care for their bodies if they die [16]. The report further referenced data available in August 2014 at Guinea’s Ministry of Health that 60% of cases in that country could be linked to traditional burial and funeral practices. The same report indicated that as at November 2014, 80% of cases estimated in Sierra Leone were linked to these practices [16]. Similarly other studies have also indicated that economic, linguistic, familial, health-seeking and other factors influence the complexity of cross-border interactions thereby challenging national systems’ capacities to detect public health events among these mobile populations including the undermining of a top-down approach to trans-border epidemic control [2,3,6]. Therefore understanding the social and cultural or traditional environment of communities and their interactions along the border as participants intimated and suggested in this study will go a long way towards effective disease control especially when packaging health educational and promotional messages.

Seeking health care across the border was one prominent finding of this study as all key informants and majority of the focus group discussants identified it to be one of the main cross-border interactive activities. Whereas those from Burkina Faso seeking health care in Ghana are likely to receive education and other public health interventions because they visit conventional health institutions in Ghana, the same cannot be said of Ghanaians who seek health care in Burkina Faso because most of them visit traditional healers and soothsayers whom themselves are not adequately equipped with the necessary knowledge and skills of disease outbreaks. This is a potential target area for improvement by agencies collaborating on disease surveillance and control.

The West African Ebola outbreak experience provides sufficient basis to place communities at the center of all intended actions to avert the Ebola virus spread to untouched communities. In this study, community members themselves together with key informants provided a wide spectrum of solutions to control the Ebola outbreak which provides an entry point for involving communities in the design and implementation of preventive measures against similar public health challenges. Our findings are also similar to that of a study to respond to communicable diseases in internationally mobile populations at points of entry and along porous borders in Nigeria, Benin and Togo where all the participants in a qualitative study agreed that good collaborative relationship between local authorities on both sides of the border was key to all other technical coordination and collaboration [5].

Strengthening individual, community and institutional capacity was suggested by both key informants and focus group discussants as one of the key solutions to effective Ebola control. Building and strengthening capacity through the training and retraining of local community actors especially health workers and those perceived to be involved in disease surveillance and control was widely expressed by study participants. Participants recommended that these local community actors from both sides of the border receive same training. This was also the view of all participants in a qualitative study involving Nigeria, Benin and Togo when they advocated that training curricula and other materials must be developed specifically for target trainees at borders [5].

## Limitations

Despite the positives of this study, it was not without limitations. First, as is usual of every qualitative study, the findings cannot be said to be representative of the entire population. Second, although there are other contiguous communities in the study area, this study concentrated on only six conveniently predetermined communities therefore leaving the possibility of extra valuable information not being captured. The last is that although the composition of the FGDs was heterogeneous, group members were mainly elders and representatives of the communities and therefore their opinions are not necessarily the views of the ordinary members of the communities especially adolescents since they are hardly opinion leaders or representatives of communities.

## Conclusion

This qualitative study that carried out forty six KIIs and six FGDs found social interactions, traditional and religious practices, seeking health care and informal trade to be the main existing interactions amongst frontline or contiguous border communities which had implications for Ebola outbreak and future pandemics. Solutions suggested by local community members and other key stakeholders to effective control of Ebola included collaborative efforts, capacity strengthening, health education and promotion from both countries. We therefore recommend that, in order to build strong and resilient health systems that can deal with the Ebola scare and other future pandemics in the border communities of Ghana and Burkina Faso and sub-Saharan African in general, stakeholders need to understand the nature of these interactions in such communities. We also recommend to stakeholders involved in global public health interventions that, seeking locally proposed solutions such as found in this study will be very useful.

## Data Accessibility Statement

All relevant data are within this manuscript. However, extra information can be requested for from the corresponding author.
